# Clinical Utility of Respiratory Scores at Admission for Estimating the Definitive Microbiological Diagnosis in Lower Respiratory Tract Infections in Infants

**DOI:** 10.1177/2333794X221098830

**Published:** 2022-06-27

**Authors:** And Demir, Nihal Özdemir Karadas, Ulas Karadas

**Affiliations:** 1 University of Helsinki and Helsinki University Hospital, Helsinki, Finland; 2 Ege University Faculty of Medicine, İzmir, Türkiye; 3 University of Health Sciences, İzmir, Türkiye

**Keywords:** viral, bacterial, infection, hospitalization, lower respiratory tract

## Abstract

We aimed at determining the clinical utility of respiratory scores and the durations of wheezing or respiratory distress during hospitalization in infants with lower respiratory tract infections (LRTI) at admission for estimating the definitive microbiological diagnosis. We obtained data from a study population of 201 patients, 79 girls and 122 boys. There was a significant divide in the causative agents of LRTI among patients younger and older than 6 months of age (*P* = .002), and significantly different respiratory score findings were determined in infants with viral LRTI: a low respiratory score in a younger-than-6 month infant suggests Adenovirus as the causative agent and a high respiratory score suggests Parainfluenza 1 or 2; as for infants of 6 months of age or older, a low respiratory score indicates Influenza A or B or a mixed infection, whereas a high respiratory score is likely an indication of Parainfluenza 3 or RSV.

## Introduction

The most common cause of morbidity and hospitalization in infants is respiratory tract infections.^1^ The prevalence of respiratory infections during infancy was shown to drop below 5% but also rise above 30% depending on the season and other factors according to various studies on this matter.^2^ UNICEF annual report 2019 has revealed additional information on young children in Türkiye. The survey results report the infant mortality rate to be at 9.3 per thousand live births in 2018.^3^ These data show that lower respiratory tract infections (LRTI) especially in infants is still a major public health problem leading to high mortality and morbidity. Therefore, it becomes increasingly important to determine the epidemiological characteristics, natural development, clinical course, risk factors, as well as appropriate diagnosis and treatment of LRTI. It is of great importance to prepare guidelines containing empiric treatment recommendations for physicians working in all walks of disciplines, taking into account the socio-economic conditions and epidemiological data at hand.^3^ It is recommended to take a blood culture before starting treatment in patients with pneumonia with fever who need to be followed up in the hospital. It is recommended to take a blood culture in presumably pneumonia patients with fever preferably immediately on the day of hospital admission before starting treatment of any sort^3^; however, blood cultures are rarely positive in pneumonia.^4^ At most half of the causative agents behind childhood pneumonia can be identified from the epidemiological aspect by conventional, serological, and molecular diagnostic methods.^4,5^ Currently, LRTI is known to be caused by over 300 agents, of which approximately 75% are viruses.^5,6^ Indeed, viral respiratory infections emerge as the most common cause for community-acquired pneumonia associated with hospitalization in the pediatric age range.^7^

In childhood pneumonia from an epidemiological point of view, only 40% to 50% of the factors can be determined using blood culture, serological and molecular biological methods in accordance with good laboratory practice (GLP) principles.^8,9^ Increasing bacterial and viral isolation studies, knowing the most common factors by age will provide the opportunity to initiate appropriate treatment options immediately.

In this study, we have addressed issues pertaining to the association of causative agents with specific clinical, laboratory, and radiological findings and severity level of the clinical picture (such as respiratory score, respiratory distress, wheezing) as well as differential diagnosis as an overall goal. We aimed at determining the clinical utility of respiratory scores in infants with LRTI at admission for estimating the definitive microbiological diagnosis, which in turn was evaluated as a tool to estimate the clinical prognosis such as durations of wheezing or respiratory distress during hospitalization.

## Materials and Methods

We performed a prospective observational study in infants with LRTI and collected data through clinical examination, basic laboratory and radiological studies, determination of causative bacterial or viral agents as well as recording associations or differences of various treatment modalities, respiratory scores or durations of wheezing and hospital stay with the underlying clinical diagnosis.

### Patients and Samples

Patients (aged between 1 and 12 months) hospitalized at Department of Pediatric Infectious Diseases, Dr. Behçet Uz Children’s Hospital University of Health Sciences (İzmir, Türkiye) due to LRTI (acute bronchiolitis, pneumonia or bronchilitis combined with bronchopneumonia) from January through April have been grouped according to clinical and radiological findings at admission and later investigated for several parameters as outlined below.

All patients with LRTI conformed with the inclusion criteria of the study. The diagnosis of bronchiolitis was based primarily on the history of illness and physical examination findings.^10^ Chronic diseases (severe malnutrition, congenital heart diseases, chronic respiratory system diseases, diabetes, chronic kidney disease, muscle and nervous system diseases, metabolic diseases) and immunodeficiency were the main exclusion criteria of the study. Demographic features were determined by taking detailed information at the time of admission. The following information was recorded as a history record for each patient: the date of onset of complaints, attendance in nursery, presence of wheezing, number of attacks, the vaccination scheme and presence of chronic and allergic disease. Any history of disease during the neonatal period was also noted. Detailed physical examinations of all cases were performed. Finally, detailed information about family and ancestors was also recorded. Durations of wheezing or respiratory distress were recorded prospectively. Periods of respiratory distress and wheezing were divided into 3 groups: under 2 days, 2 to 5 days, 6 days and more.

Patients were given respiratory scores. The necessity of referral and admission for hospitalization were determined by respiratory scoring based on clinical evaluation in bronchiolitis.^11,12^ Mild disease (1-3 points): breaths per minute (BPM) was below 45; the ronchi could be heard only by stethoscope and at the end of expirium; there was no intercostal retraction. Moderate disease (4-8 points): BPM 45-60; wheezing could be heard in expirium without stethoscope. The patient exhibited intercostal retractions; there was no cyanosis. Severe disease (9-12 points): BPM 60; patient presented with wheezing, which could be heard without a stethoscope. The patient had cyanosis, dehydration and severe respiratory distress. If the patient had apnea, it was considered severe regardless of scoring.

### Laboratory Studies

Blood was drawn from each patient in order to identify the bacterial agent in cultures and to show possible bacteremia. Serum CRP was considered positive at 1 mg/dL and above, negative when the serum CRP level was below 5 mg/L, and serum CRP levels were determined to be mildly (5-10 mg/L), moderately (11-50 mg/L), heavily (51-150 mg/L), and very heavily (>150 mg/L) elevated for respective values. An anteroposterior X-ray, and if so required also a lateral one was taken from each case. Lung CT was taken only in patients if clinical and laboratory findings warranted so. An hourly red blood cell sedimentation rate higher than 20 mm/hour and a white blood cell count below 5000/mL or above 14 000/mL were regarded as pathological.

ELISA screening kits based on a single reagent set system for all virus parameters (Clinikit and DiagnoPack, Orgenium Laboratories Oy, Finland) were used for the detection of IgG and IgM antibodies against designated viruses being Adenovirus, Influenza A, Influenza B, Parainfluenza 1, Parainfluenza 2, Parainfluenza 3, and Respiratory syncytial virus (RSV). A 2-mL of blood sample was taken from all cases at the time of hospitalization, and another one in 2 to 3 weeks for detecting possible seroconversion. The blood samples were centrifuged on the same day of collection and the serum sample on the supernata was placed in Eppendorf (Hamburg, Germany) tubes and frozen at −80°C. The presence of IgM antibodies or 4 times or more increase in IgG level was evaluated as signs of a positive infection.

IgM and IgG antibody titers for Herpes Simplex Virus 1 (HSV-1) and HSV-2, Coxsackievirus B5, Echovirus, Enterovirus, Cytomegalovirus were investigated for possible cross-reactions. High titer results in Influenzae B and Parainfluenzae 1-2-3 specific IgMs were regarded as potential secondary reactions to Coxsackievirus B5, Echovirus, or Enterovirus infections as per the instructions provided by the manufacturer of the kits.

### Statistical Analyses

Parametric values were expressed as average ± standard deviation. The Pearson Chi-Square and binomial tests were used along with Phi and Cramer’s V data for comparisons of arguments. A *P*-value less than .05 was considered statistically significant. The Mann-Whitney *U* and Kruskal-Wallis tests were utilized for the nonparametric comparison of data from either 2 or more groups, respectively.

### Ethical Approval and Informed Consent

Ethics committee approval (BUCHVCEAH-180105-25-06) was obtained from Dr. Behçet Uz Children’s Hospital, University of Health Sciences, İzmir, Türkiye before starting with the study. The study has complied with the World Medical Association Declaration of Helsinki regarding ethical conduct of research involving human subjects. During the study, all parents or custodians were notified with an information leaflet with details about the research study, and all of them have provided written consent before their patients were admitted to the study.

## Results

At the end of the study period, we obtained data from a study population of 201 patients, 79 girls and 122 boys.

### Clinical Findings

Auscultation findings at admission were used for preliminary classification of cases into diagnostic groups as follows: those with rales only were classified as pneumonia (n = 23, 11.4%) and those with wheezing as bronchiolitis (n = 69, 34.3%); the patients who presented with both rales and wheezing were regarded as a combination of pneumonia and bronchiolitis (n = 105, 52.2%) (Table 1, panel A). According to initial clinical assessment, mean ages of the patients with pneumonia, bronchiolitis or a combination of bronchiolitis and bronchopneumonia were not statistically different. There were no cases with clinical findings associated with empyema, pleurisy, pneumopleurisy or pneumonia plus empyema.

**Table 1.** Mean Age of Patient Groups With Various Infections According to Clinical Assessment (Panel A) and Microbiological Analysis (Panel B).

A	n	%	Mean age (months)	SD
Pneumonia	23	11.4	7.6	1.8
Bronchiolitis	69	34.3	6.3	2.1
Pneumonia + bronchiolitis	105	52.2	6.3	3.8

B	Adenovirus	Influenza A	Influenza B	Parainfluenza 1	Parainfluenza 2	Parainfluenza 3	RSV	Mixed infection
Mean age (months, *P* = .002)	4.0	8.0	8.5	3.6	5.0	7.5	8.2	7.9

Mean ages of patients infected by various pathogens detected in the study population were compared. Mean ages of the patients who tested positive for Influenza A, Influenza B, Parainfluenza 3, and RSV were significantly higher than those infected by Adenovirus, Parainfluenza 1, and Parainfluenza 2 (*P* = .002, Table 1, panel B).

Respiratory distress disappeared within the first 2 days in 77.1% of the cases (n = 155). The rate of disappearance of wheezing within 2 days remained at 14.4% (n = 29). Wheezing continued for 6 days or more in 26.9% of the cases (n = 54) and respiratory distress in only 2.5% of the cases (n = 5). Investigations in regard to the etiological aspect of the 5 cases with respiratory distress lasting for at least 6 days revealed 2 cases with viral infections due to a single agent (Influenza A and Parainfluenza 3); no causative agent could be identified in the other 3 cases. The patients with persistent wheezing for at least 6 days were confirmed to be infected with Influenza A (20%), Influenza B (16%), or mixed infections (10%).

The patients with respiratory scores 4 to 8 and 9 to 12 given at admission to hospital were compared in terms of duration of hospitalization, respiratory distress and wheezing. Durations of hospital stay or respiratory distress was not any different among groups with respiratory scores of 4 to 8 and 9 to 12 (*P* = .22 and *P* = .42, respectively). On the other hand, duration of wheezing, which was an inherent factor in determining respiratory scores, was statistically different between the 2 groups with the above-mentioned respiratory scores obtained at the initial examination (*P* = .03).

### Basic Laboratory Findings

Despite the statistically insignificant difference (*P* = .09), the positive rate of CRP was found to be high in patients with clinical signs and symptoms of pneumonia. Also body temperature and erythrocyte sedimentation rate findings were not different enough to differentiate between different clinical diagnoses (*P* = .72 and *P* = .59, respectively). Similarly, X-ray findings alone were not sufficient to differentiate between different clinical diagnoses as expected (*P* = .09).

### Evaluation of Blood Culture Results

Blood cultures were identified with a specific bacterial agent in 3 (1.5%) of the patients who were hospitalized with LRTI. Two of the microorganisms detected in blood culture were Staphylococcus epidermidis and Staphylococcus haemolyticus, and the third identification indicated coagulase-negative staphylococci, which are among rare causes of any respiratory infection, thus regarded as a contamination. Regardless of the unclarity in definitive diagnosis, empirical antibiotics treatment was initiated in 2 of these 3 patients. Modification of the antibiotics treatment regimen was not required in any of the cases according to the results of blood culture showing no resistance to the initially selected drug of treatment.

### Evaluation of Viral Serology Results

At least 1 possible viral agent was detected in 124 (61.7%) of the 201 patients with serum samples. A total of 152 types or combinations of microorganisms were isolated in 124 patients due to mixed infections, 56 of which were identified with antibodies against Influenza B (36.8%), 46 of them against Influenza A (30.3%), 26 of them against Parainfluenza (17.1%), 15 of them against RSV (9.9%), and 9 of them against Adenoviruses (5.9%). Mixed infection was identified in 20 of the 124 cases; Influenza B was the clearly the most common causative agent (n = 18) out of the 20 mixed infection cases. Five of the mixed infections were caused by Influenza B and RSV, 4 of them by Influenza B and Adenovirus, 4 of them by Influenza B and Influenza A. The rest of the mixed infections were caused as 1 single case by a separate unique combination as follows: Influenza B plus Influenza A plus RSV, Influenza B plus Influenza A plus Parainfluenza 1, Influenza B plus Parainfluenza 1, Influenza B plus Parainfluenza 1 plus Adenovirus, Influenza B plus Parainfluenza 1 plus RSV, Influenza A plus Parainfluenza 1, Influenza A plus Parainfluenza 2.

IgM and IgG type antibodies against various viral agents was detected as positive in 61.7% of the cases (n = 92). This result is in line with those of the earlier studies in the literature. Usually a single virus is the causative agent in a pediatric LRTI case, but more than one virus can be isolated in 5% to 20%.^13^ In our study, we encountered a mixed viral infection in 13 cases (14.1%) with no bacterial involvement. Additionally, there were 2 cases with mixed viral and bacterial agents, of which one presented as a combination of Influenza B and Staphylococcus epidermidis and the other one as a combination of Influenza B, Parainfluenza 1, RSV, and coagulase-negative staphylococci. Thus, the total number of mixed infection cases was 15 (16.3%).

No Adenovirus nor any Parainfluenza 1 or 2 viruses were found in any of the cases of pneumonia, and there was no mixed-type causative agent in cases of pneumonia (Table 2, panels A and B). All mixing agents were found to be causative agents in patients diagnosed with bronchiolitis and bronchopneumonia + bronchiolitis (Table 2, panel A). Microbiological agents identified herein were associated with the clinical findings depicted in Table 3. Quantitative values of CRP and body temperature were compared among different groups of infection and no significant relationship was found (*P* = .29 and *P* = .34, respectively) (Table 3). However, significantly different respiratory score findings were determined in patients whose LRTI was caused by different groups of pathogens as identified in this study. Respiratory scores were significantly lower in those infected with Adenovirus, Influenza A, Influenza B or mixed agents, and significantly higher in those infected with Parainfluenza 1, Parainfluenza 2, Parainfluenza 3, and RSV (*P* = .02 overall, *P* < .05 for individual pathogens) (Table 3). Unlike the case for respiratory scores, there was no significant difference between the identified microbiological agents and durations of respiratory distress or wheezing (*P* = .56 and *P* = .62, respectively) (*P* values .56 and .62, respectively) (Table 4). Also, there was no significant relationship between the identified microbiological agents and X-ray findings (normal, hyperlucensy. pulmonary infiltrate, hyperlucensy + pneumonic infiltrate) or blood culture results (negative, positive, contamination) (*P* = .12 and *P* = .64, respectively).

**Table 2.** Number and Percentages of Various Types of Lower Respiratory Tract Infections for Each Causative Viral Agents (Panel A); Number and Percentages of the Causative Viral Agents in Various Cases of Lower Respiratory Tract Infections (Panel B).

Panel A	n	Bronchiolitis	%	Pneumonia	%	Pneumonia + Bronchiolitis	%
Pathogen involved		n		n		n	
Adenovirus	9	1	11.1	0	0.0	8	88.9
Influenza A	46	15	32.6	6	13.0	25	54.3
Influenza B	56	21	37.5	6	10.7	29	51.8
Parainfluenza 1	6	0	0.0	0	0.0	6	100.0
Parainfluenza 2	10	4	40.0	0	0.0	6	60.0
Parainfluenza 3	10	2	20.0	0	0.0	8	80.0
RSV	15	0	0.0	3	20.0	12	80.0
Mixed infection	20	8	40.0	0	0.0	12	60.0

Panel B	Bronchiolitis		Pneumonia		Pneumonia + Bronchiolitis	
Pathogen	n	%	n	%	n	%
	n = 41		n = 10		n = 73	
Adenovirus	0	0.0	0	0.0	3	4.1
Influenza A	11	26.8	4	40.0	20	27.4
Influenza B	17	41.5	4	40.0	23	31.5
Parainfluenza 1	0	0.0	0	0.0	3	4.1
Parainfluenza 2	3	7.3	0	0.0	3	4.1
Parainfluenza 3	1	2.4	1	10.0	4	5.5
RSV	0	0.0	1	10.0	4	5.5
Mixed infection	9	22.0	0	0.0	13	17.8

**Table 3.** Comparison of CRP Values, Body Temperature, and Respiratory Scores Among the Viral Pathogens Causing LRTI in Infants.

	Adenovirus		Influenza A		Influenza B		Parainfluenza 1, 2, 3		RSV		Mixed infection	
	n	%	n	%	n	%	n	%	n	%	n	%
CRP (mg/dL, *P* = .29)												
0-1	2	40.0	33	57.9	14	19.7	18	78.3	5	55.6	18	51.4
1-5	3	60.0	20	35.1	48	67.6	3	13.0	1	11.1	12	34.3
≥5.1	0	0.0	4	7.0	9	12.7	2	8.7	3	33.3	5	14.3
Body temperature (°C, *P* = .26)												
36.0-37.8	3	60.0	52	91.2	55	77.5	16	69.6	7	77.8	31	88.6
37.9-39.0	1	20.0	1	1.8	12	16.9	7	30.4	2	22.2	4	11.4
39.1-40	1	20.0	4	7.0	4	5.6	0	0.0	0	0.0	0	0.0
≥40	0	0.0	0	0.0	0	0.0	0	0.0	0	0.0	0	0.0
Respiratory score (points, *P* = .02)												
4-8	5	100.0	39	68.4	51	71.8	6	26.1	3	33.3	32	91.4
9-12	0	0.0	18	31.6	20	28.2	17	73.9	6	66.7	3	8.6

**Table 4.** Comparison of the Duration of Wheezing and RD With the Identified Pathogen.

	Adenovirus		Influenza A		Influenza B		Parainfluenza 1, 2, 3		RSV		Mixed infection	
	n	%	n	%	n	%	n	%	n	%	n	%
Duration of wheezing, (days, *P* = .56)												
<2	2	40.0	5	8.8	7	9.9	1	4.3	5	55.6	0	0.0
2-5	3	60.0	34	59.6	46	64.8	15	65.2	2	22.2	24	68.6
≥6	0	0.0	18	31.6	18	25.4	7	30.4	2	22.2	11	31.4
Duration of respiratory distress (days, *P* = .62)												
<2	3	60.0	48	84.2	62	87.3	20	87.0	7	77.8	28	80.0
2-5	2	40.0	7	12.3	9	12.7	1	4.3	2	22.2	7	20.0
≥6	0	0.0	2	3.5	0	0.0	2	8.7	0	0.0	0	0.0

No occurrence of transfer to pediatric intensive unit or death was noted in this study.

## Discussion

Distinguishing viral infections from bacterial and mixed infections as well as differential diagnosis of viral infections are demanding tasks in most children.^14,15^ Of all pneumonia instances, 7% to 13% were severe enough to be life-threatening and necessary hospitalization.^14^ LRTI presents mainly in 2 clinical forms, pneumonia and bronchiolitis.^16^ Differential diagnosis of these 2 is another challenging issue.^17^ Although the incidence of LRTI is at comparable levels worldwide,^18^ morbidity and mortality rates are up to 15 times higher in developing nations. In 2010, over 10 million pneumonia cases were reported worldwide, approximately 3 million of them were severe cases and 1.4 million of them ended up in exitus.^19^ Higher mortality rates have been reported to be associated with risk factors like young age (particularly <1 year) and malnutrition.^20^

In addition to the above, our results confirm the challenge in differential diagnosis of viral LRTIs by clinical examination in addition to basic laboratory and radiological findings. As for pneumonia patients with fever requiring monitorization at a hospital, a blood culture is definitely recommended before starting treatment, but in pneumonia, blood cultures are rarely positive.^4^ In a vast majority of the cases, organisms detected by blood culture, there was no resistance to empiric treatment, which was initially selected. Again findings of our study revealed a positive result in only 3.3% of the blood culture samples taken due to a preliminary diagnosis of pneumonia. There was no justified need to change the choice of initial antibiotics based on blood culture results in any of the cases of our study, which was in accordance with the earlier findings reported in the literature.^21^ Also CRP and sedimentation rate values have not provided sufficient information in differentiating viral and bacterial pneumonia in this study. Many prospective studies have shown that acute phase reactants have low sensitivity and specificity in differentiating viral and bacterial or combined infection.^22^ Accordingly, excessively high sedimentation and CRP values are known to support the diagnosis of bacterial infection^23,24^; these basic laboratory findings were quite distinct but no such significant difference to serve differential diagnosis was found in this study (*P* = .09). Therefore, no single determining factor could be demonstrated for differentiating the causative agents of LRTI in infants.

Interestingly however, we have demonstrated that the causative agent of an LRTI in an infant can be preliminarily estimated based on a simple algorithm of respiratory score and age of infant with LRTI as follows: a low respiratory score in a younger-than-6 month infant suggests Adenovirus as the causative agent and a high respiratory score suggests Parainfluenza 1 or 2; as for infants of 6 months of age or older, a low respiratory score indicates Influenza A or B or a mixed infection, whereas a high respiratory score is likely an indication of Parainfluenza 3 or RSV (Table 5).

**Table 5.** Preliminary Assessment of the Causative Agent in an Infant With Viral LRTI Based on a Simple Algorithm of Respiratory Score and Age (Data Derived From Tables 1 and 3).

	Respiratory score	
	4-8	9-12
Age <6 months	Adenovirus	Parainfluenza 1
		Parainfluenza 2
Age ≥6 months	Influenza A	Parainfluenza 3
	Influenza B	RSV
	Mixed infection	

We can conclude that respiratory score can be utilized as a triage tool for preliminary identification of possible causative agents in infants with viral LRTI, before confirming or rejecting the preliminary results via definitive diagnostic processes.
